# Should we educate about the risks of medication overuse headache?

**DOI:** 10.1186/1129-2377-15-10

**Published:** 2014-02-13

**Authors:** James TF Lai, John DC Dereix, Ravi P Ganepola, Peter G Nightingale, Kiera A Markey, Paul N Aveyard, Alexandra J Sinclair

**Affiliations:** 1 Department of Medical Education, University of Birmingham, Birmingham, UK; 2 Wellcome Trust Clinical Research Facility, Queen Elizabeth Hospital, Birmingham, UK; 3 Department of Primary Care Health Sciences, University of Oxford, Oxford, UK; 4 Neurotrauma and Neurodegeneration, School of Clinical and Experimental Medicine, College of Medical and Dental Sciences, University of Birmingham, Birmingham B15 2TT, UK

**Keywords:** Medication-overuse headache, Analgesia, Headache, Prevention, Education

## Abstract

**Background:**

Medication-overuse headache (MOH) is caused by the regular use of medications to treat headache. There has been a lack of research into awareness of MOH. We distributed an electronic survey to undergraduate students and their contacts via social networking sites. Analgesic use, awareness of MOH, perceived change in behaviour following educational intervention about the risks of MOH and preferred terminology for MOH was evaluated.

**Findings:**

485 respondents completed the questionnaire (41% having received healthcare training). 77% were unaware of the possibility of MOH resulting from regular analgesic use for headache. Following education about MOH, 80% stated they would reduce analgesic consumption or seek medical advice. 83% indicated that over the counter analgesia should carry a warning of MOH. The preferred terminology for MOH was painkiller-induced headache.

**Conclusions:**

This study highlights the lack of awareness of MOH. Improved education about MOH and informative packaging of analgesics, highlighting the risks in preferred lay terminology (i.e. painkiller-induced headache), may reduce this iatrogenic morbidity and warrants further evaluation.

## Background

Medication-overuse headache (MOH) is a form of chronic daily headache. Its diagnosis is based on the patients history of headache present on 15 or more days per month with use of regular acute and/or symptomatic headache treatment for at least 3 months [1,2]. The definition of overuse varies depending on the medication involved and is defined in terms of both duration of use and the number of treatment days per month [1]. MOH can be a chronically disabling condition and may have a greater impact on daily function than episodic migraine [3]. Previous studies have demonstrated a worldwide prevalence of MOH ranging from 0.9% to 1.8% [4-8]. Recognition of MOH is key to improving headache disability and responsiveness to headache prophylactic drugs [9]. Hagen *et al.* identified risk factors for MOH in which a headache frequency of 7–14 days per month was strongly associated [10]. All acute medications used to treat headache are capable of causing MOH. Triptans are the most frequently used drug by patients who develop MOH [11]. The mechanisms underlying MOH are unknown. However, the number of analgesic free days, rather than the absolute amount of medication used, is felt to be key in the development of MOH [2,11]. There has been no previous research looking at the awareness of MOH or the potential of educational preventative strategies in MOH. Our study aimed to investigate the awareness of MOH amongst a network of social media users derived from undergraduate students. Their self-predicted behaviour modifications following education about MOH and opinions towards warning labels on analgesic packaging was evaluated. Finally, they identified their preferred terminology for MOH.

## Methods

This project did not involve NHS patients or staff. It was reviewed by the University of Birmingham and permission was granted to distribute the questionnaire amongst the student population and their social media connections.

Undergraduate students at the University of Birmingham, UK were approached by email or via social networking sites and invited to complete an online questionnaire. They were further requested to disseminate the questionnaire to their social media network, consequently it was not possible to quantify uptake rates. Questions were worded for a lay audience and answers were self reported by the participant from a selection of drop-down boxes (see Additional file 1: Table S1). All completed questionnaires were included for analysis. Participants’ demographics, education and previous healthcare training were recorded. Healthcare training was self reported and included medical and healthcare professionals and trainees. Education level was defined by the highest qualification received at secondary (GCSEs or equivalent), further (A levels or equivalent) or higher (diploma, degree, masters) education. The type of analgesia used and the reason for its use was documented along with awareness of side effects (from a choice of well described as well as irrelevant options). Participants were then asked to select the type of pain they were treating. Those who reported treating headache were subsequently asked about frequency of analgesic use and number of headache days over the previous month to ascertain those with MOH.

All responders were educated about the possibility of developing MOH as a consequence of regular analgesic use. We asked participants if and how this would alter their behaviour (stop or reduce their use of analgesics, or seek medical advice). Participants were then asked to select their preferred label for MOH (see Additional file 1: Table S1). Finally, we asked respondents whether they thought painkiller packaging should carry a warning message cautioning users of the risk of MOH. Statistical analysis was performed with PASW Statistics for Windows version 18.0 (SPSS Inc., Chicago, Illinois). Data were summarised using counts and percentages for categorical data or medians and ranges for continuous data. Comparisons of two groups were made using Fisher’s exact test or the Mann–Whitney test. The level of significance was set at P < 0.05. A binary logistic regression analysis was performed with MOH awareness as the dependent variable and age, gender and healthcare training as the explanatory variables.

## Findings

485 people completed the questionnaire. Responders were divided into those that had received healthcare education and those who had not. Participant demographics are shown in Table 1. Paracetamol was the most commonly used analgesic (379 individuals; 78%) (Additional file 2: Figure 1). Apart from the use of aspirin, which was significantly lower in the healthcare educated group (P = 0.032), our data demonstrate no significant differences in types of medication used between our groups.

**Table 1:** Demographics of questionnaire responders

	**Study population N = 485 (%)**	**Healthcare training N =197 (%)**	**No healthcare training N =288 (%)**
**Gender**			
Male	179 (37)	48 (24)***	131 (46)***
Female	305 (63)	149 (76)***	156 (54)***
**Age** (median; range)	23 (12–99)	22 (18–99)***	29 (12–88)***
**UK national**	466 (96)	192 (97)	274 (95)
**Ethnicity**			
White	396 (82)	160 (81)	236 (82)
Asian	48 (10)	25 (13)	23 (8)
Black	10 (2)	4 (2)	6 (2)
Mixed	9 (2)	4 (2)	5 (2)
Chinese	20 (4)	4 (2)	16 (6)
No response	2 (0)	0	2 (1)
**Education level**			
Secondary	477 (98)	197 (100)	280 (97)
Further	443 (91)	191 (97)	252 (88)
Higher	320 (66)	133 (68)	187 (65)

Figures are expressed as means and percentages unless stated otherwise. ***indicates significant difference between healthcare training and non-healthcare training groups at a level of P < 0.001.

**Figure 1:**
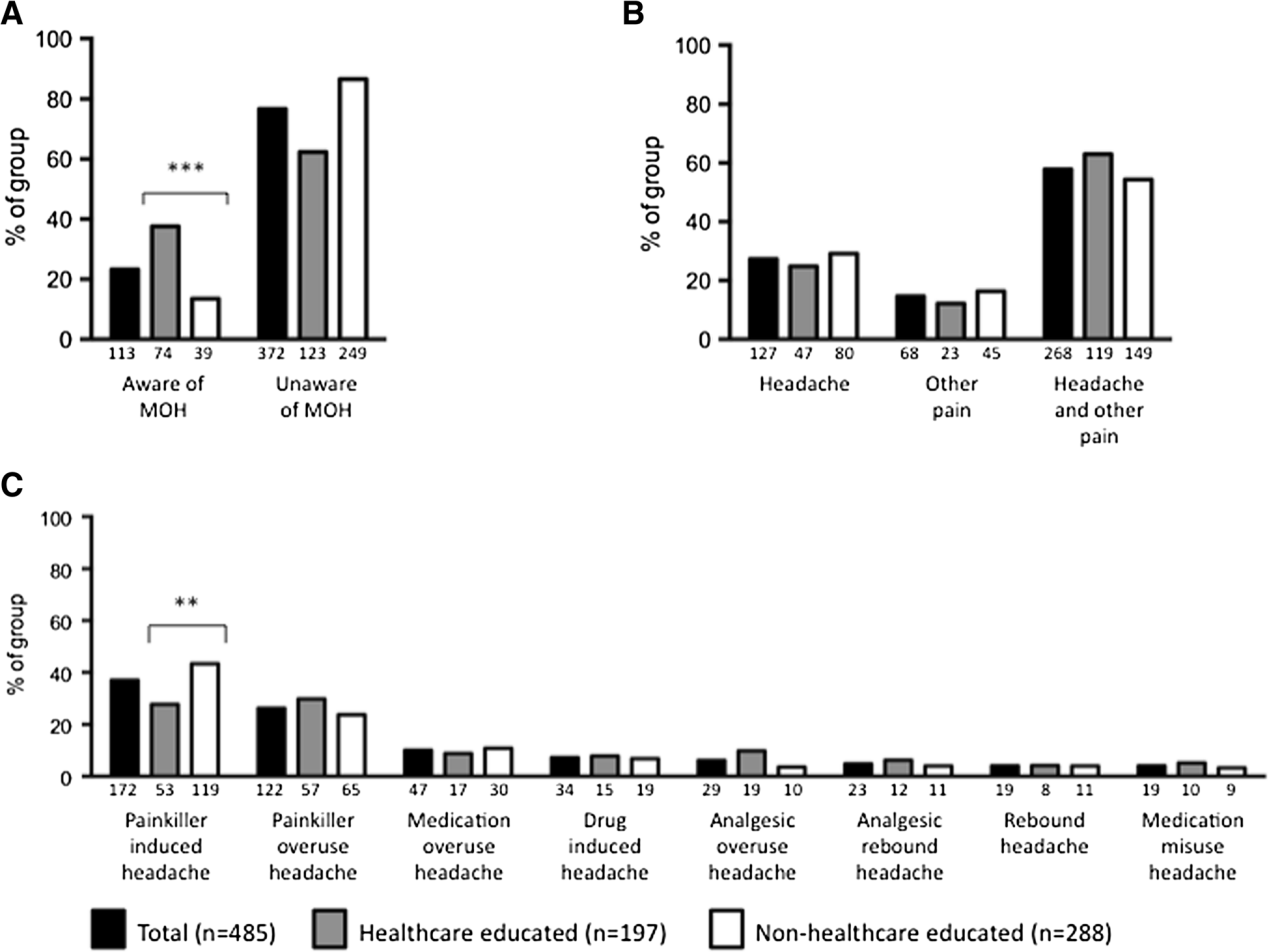
**Awareness, reason for using analgesia and preferred nomenclature for medication overuse headache. A)** Awareness of MOH within our sample. **B)** Reason for analgesic use. **C)** Preferred name for medication overuse headache. Respondents consisted of healthcare educated (n = 197; grey bars) and non-healthcare educated (n = 288; white bars) individuals. Data are demonstrated as percentage of sample selected with the absolute value below each bar. **indicates p < 0.01 and ***indicates p < 0.001.

Awareness of MOH as a side effect of regular analgesic use was noted in just 113 (23%) individuals, 74 (38%) in the healthcare educated group and 39 (14%) in the non-healthcare educated group (p < 0.001, Figure 1A).The difference between the groups was significant (p < 0.001) in a multivariable analysis adjusting for age (p = 0.56) and gender (p = 0.82). As would be expected, the identification of potential drug side effects differed significantly between the healthcare and non-healthcare educated groups, with the healthcare group identifying the correct side-effects more frequently.

Analysis of the whole cohort revealed that analgesic use was predominantly for headaches (395 individuals; 85%). This consisted of individuals using medication for isolated headache (127; 27%) and for headache in conjunction with other pain (268; 58%) (Figure 1B). 35 (7%) had greater than 14 headache days per month and 11 (2%) have MOH. Of those using analgesia for headaches, most used analgesia 1–3 days per week (94; 24%), while 12 (3%) used analgesia 4–6 days per week and 4 (1%) on a daily basis. The majority (336; 85%) had not sought medical advice for their headache. Interestingly, evaluation of the preferred nomenclature for MOH revealed that healthcare educated individuals favoured the term painkiller overuse headache (53; 27%). However, non-healthcare educated respondents preferred the term painkiller-induced headache (119; 43%), which was also favoured overall (172; 37%). The current term of MOH was favoured by only 47 individuals (10%; Figure 1C).

**Figure 2:**
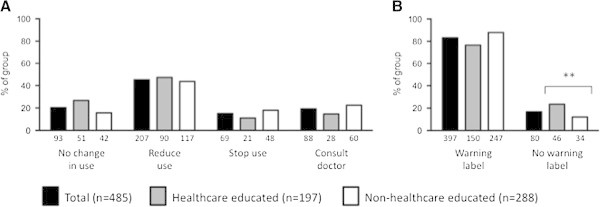
**Behavioural changes and warning label preferences following education about medication overuse headache. A)** Change in behaviour indicated following education about medication overuse headache. **B)** Preference for a warning label about medication overuse headache to be added to packaging. Healthcare educated (n = 197; grey bars) and non-healthcare educated (n = 288; white bars) individuals. Data are demonstrated as percentage of sample selected with the absolute value below each bar. **indicates p < 0.01.

Following education about the risk of MOH, the majority (364; 80%) of participants indicated they would alter their analgesic taking behaviour. Of these, 207 (57%) would reduce their usage, 69 (19%) would stop using their analgesia and 88 (24%) would consult a doctor for advice on headache treatment (see Figure 2A). Healthcare educated individuals were significantly more likely to suggest that they would not alter their analgesic use (healthcare education 51 (27%) vs. non-healthcare educated 42 (16%); P = 0.005). Figure 2B demonstrates that the majority of our sample (397; 83%) would advocate a written warning on the packaging of analgesics explaining the risk of MOH. There was a greater proportion of healthcare educated responders amongst those indicating their opposition to a warning label about MOH (healthcare educated 46 (23%) vs. non-healthcare educated group 34 (12%); P = 0.002).

## Discussion

There have been no previous studies looking at the awareness of MOH and this was an area requiring further investigation. Our study population consisted of individuals with and without healthcare training and, as all participants were identified from the social media network of University of Birmingham students, there is likely to be significant responder bias towards a higher educated population. We would predict, therefore, that the results in the general population would highlight even less education and knowledge of MOH.

Our results highlight that participants had limited knowledge of MOH. The majority of our population would change their analgesic usage if they knew about the risk of MOH and supported the idea of a warning label on packaging. Our results suggest that warning labels on packaging may have a considerable impact on the prevention of MOH. Work by Grand *et al.* supports this theory, their study involved delivering brief intervention in the form of short, verbal information and a neurological examination to patients with chronic headache. Both chronic headache and medication overuse were reduced following their intervention [12]. The most popular name for MOH was “painkiller-induced headache” and future literature aimed at educating the public may be better understood if worded in these terms.

Our study was the first to investigate awareness of MOH. However, our sample was subject to responder bias due to the collection method employed and consequently we are unable to assume that the results reflect those of the general population, in fact, it is likely that knowledge of MOH may have been overestimated in our relatively educated cohort of responders. Thus, the issues of lack of awareness of MOH may be a larger issue than is highlighted in this small study. However, the use of closed questions and multiple-choice responses could have led to an overestimation in identifying awareness of MOH. Analgesic use may be underestimated since our study population was skewed towards the young and consequently less likely to use analgesia owing to a decreased occurrence of co-morbidities in this group. The pragmatic approach to the study meant that healthcare training, headache characteristics and medication intake were self-reported and not formally verified. The use of an online-based questionnaire was a cost effective way to recruit a relatively large sample size. A further strength was in the speed with which responses could be gathered and analysed. The use of social networking sites in the distribution of these questionnaires may be a useful tool for future research, particularly if deployed though hospital trust social media and twitter feeds. However, the responses reflect the views of those willing to complete the questionnaire and may not accurately represent the general population. Our methodology also meant that it was not possible to calculate uptake rates.

Our study identifies the need to carry out a long-term study evaluating the efficacy of a preventative strategy to reduce MOH. Furthermore, the conversion of predicted to actual changes in behaviour need evaluating. In summary, there is a lack of awareness of MOH and our figures likely underestimate the issue. Information regarding MOH may alter behaviour and interventions, such as altering the packaging of medications, may help reduce morbidity from MOH. Finally, our responders indicated that changing the name of MOH to pain-killer induced headaches would be preferable.

## Competing interests

The authors declare that they have no competing interests.

## Authors' contributions

AJS conceived the project and supervised the manuscript. JTFL, JDCD and RPG were responsible for data collection and drafted the final manuscript. PGN was responsible for statistical analysis. PNA and KAM supervised the manuscript. All authors read and approved the final manuscript.

## Authors' information

Dr Alexandra Sinclair is a NIHR Clinician Scientist.

## Supplementary Material

Additional file 1: Table S1 Questions issued to participants to complete. Click here for file

Additional file 2: Figure S1 Medication usage between the two groups demonstrates no significant changes apart from the use of aspirin. Respondents consisted of healthcare educated (n = 197; grey bars) and non-healthcare educated (n = 288; white bars) individuals. Data are demonstrated as percentage of sample selected with the absolute value below each bar. *Indicates p < 0.05. Click here for file
