# Supplementary material for: Should we educate about the risks of medication overuse headache?
Source: J Headache Pain. 2014 Feb 13;15(1):10. doi: 10.1186/1129-2377-15-10 (PMC3942071; doi:10.1186/1129-2377-15-10)
Supplement: Additional file 1: Table S1 — Questions issued to participants to complete. [file 1129-2377-15-10-S1.docx]

**Supplementary Table 1.**

| **1. Are you male or female?** | | | | | | | | | | | | | | | | | | | | | | | | | | | | | | | | | | | | | | | | | | | | | |
| --- | --- | --- | --- | --- | --- | --- | --- | --- | --- | --- | --- | --- | --- | --- | --- | --- | --- | --- | --- | --- | --- | --- | --- | --- | --- | --- | --- | --- | --- | --- | --- | --- | --- | --- | --- | --- | --- | --- | --- | --- | --- | --- | --- | --- | --- |
| Male | | Female | | | | | | | |  | | | | |  | | | | |  | | | | | |  | | | |  | | | | |  | | |  | | |  | | |  | |
| **2. What is your age?** | | | | | | | | | | | | | | | | | | | | | | | | | | | | | | | | | | | | | | | | | | | | | |
| (Open response) | | | | | | |  | | | | | | | | | | | | | | | | | | | | | | | | | | | | | | | | | | | | | | |
| **3. Which country do you live in?** | | | | | | | | | | | | | | | | | | | | | | | | | | | | | | | | | | | | | | | | | | | | | |
| (Open response) | | | | | | |  | | | | | | | | | | | | | | | | | | | | | | | | | | | | | | | | | | | | | | |
| **4. What is your ethnic identity?** | | | | | | | | | | | | | | | | | | | | | | | | | | | | | | | | | | | | | | | | | | | | | |
| White | | | Asian | | | | | | | | Black | | | | | Mixed | | | | | Chinese | | | | | | (Open response) | | | | |  | | | | | | | | | | | | | |
| **5. What is your highest level of education?** | | | | | | | | | | | | | | | | | | | | | | | | | | | | | | | | | | | | | | | | | | | | | |
| Secondary education  (GCSE, O level or equivalent) | | | | | | | | | Further education  (A level or equivalent) | | | | | | | | | | Higher education  (Diploma, degree, masters or doctorate) | | | | | | | | | | (Open response) | | | | | | | |  | | | | | | | | |
| **6. Have your received any training as a healthcare professional?** | | | | | | | | | | | | | | | | | | | | | | | | | | | | | | | | | | | | | | | | | | | | | |
| Yes | | No | | | | | | | |  | | | | |  | | | | |  | | | | | |  | | | |  | | | | |  | | |  | | |  | | |  | |
| **7. Do you use any of the following painkillers?** | | | | | | | | | | | | | | | | | | | | | | | | | | | | | | | | | | | | | | | | | | | | | |
| Aspirin | | Aspirin + codeine | | | | | | | | Diclofenac | | | | | Ibuprofen | | | | | Ibuprofen + codeine | | | | | | Ibuprofen + paracetamol | | | | Paracetamol | | | | | Paracetamol + caffeine | | | Paracetamol + codeine | | | Sumatriptan | | | I do not use painkillers | |
| **8. Please select all the side effects that you are aware of?** | | | | | | | | | | | | | | | | | | | | | | | | | | | | | | | | | | | | | | | | | | | | | |
| Headache | Bleeding | | | | | | | Kidney damage | | | | | | Liver damage | | | | Rash | | | | | Stomach ulcers | | | | | Reduced effect of contraception | | | | | | Limb swelling | | Blood clotting | | | Anorexia | | | Palpitations | | | I do not know of any side effects |
| **9. What type of pain do you use painkillers for?** | | | | | | | | | | | | | | | | | | | | | | | | | | | | | | | | | | | | | | | | | | | | | |
| Headache | | | | | Other pain | | | | | | | | Headache and other pain | | | | | | | | |  | | | | | | | | | | | | | | | | | | | | | | | |
| **10. In the last month have you had more than 14 days of headache?*** | | | | | | | | | | | | | | | | | | | | | | | | | | | | | | | | | | | | | | | | | | | | | |
| Yes | No | | | | | | |  | | | | | |  | | | |  | | | | |  | | | | |  | | | | | |  | |  | | |  | | |  | | |  |
| **11. How often have you taken painkillers because of headache in the last month?*** | | | | | | | | | | | | | | | | | | | | | | | | | | | | | | | | | | | | | | | | | | | | | |
| Seldom or never | | | | | | 1-3 days a week | | | | | | | | | | | 4-6 days a week | | | | | | | Daily | | | | | | | | |  | | | | | | | | | | | | |
| **12. Have you seen a doctor about your headache?*** | | | | | | | | | | | | | | | | | | | | | | | | | | | | | | | | | | | | | | | | | | | | | |
| Yes | No | | | | | | |  | | | | | |  | | | |  | | | | |  | | | | |  | | | | | |  | |  | | |  | | |  | | |  |
| **13. If you knew that taking painkillers on a regular basis could cause headache, what would you do?** | | | | | | | | | | | | | | | | | | | | | | | | | | | | | | | | | | | | | | | | | | | | | |
| Not change my usage | | | | | | | | | Reduce my use of painkillers | | | | | | | | | | Stop using painkillers | | | | | | | | | | Consult my doctor | | | | | | | |  | | | | | | | | |
| **14. Do you think painkillers should carry a warning message, telling users that chronic headache is a potential side effect of taking painkillers regularly?** | | | | | | | | | | | | | | | | | | | | | | | | | | | | | | | | | | | | | | | | | | | | | |
| Yes | No | | | | | | |  | | | | | |  | | | |  | | | | |  | | | | |  | | | | | |  | |  | | |  | | |  | | |  |
| **15. What do you think is the best name for headaches caused by taking painkillers on a regular basis?** | | | | | | | | | | | | | | | | | | | | | | | | | | | | | | | | | | | | | | | | | | | | | |
| Medication overuse headache | | | | Rebound headache | | | | | | | | Drug induced headache | | | | | | Medication misuse headache | | | | | | | Analgesic rebound headache | | | | | | Analgesic overuse headache | | | | | Painkiller induced headache | | | | Painkiller overuse headache | | | (Open response) | | |

* denotes questions that are only asked to individuals that used painkillers for headache or headache and other pain in question 9.
