# Supplementary figures and images for: Should we educate about the risks of medication overuse headache?
Source: J Headache Pain. 2014 Feb 13;15(1):10. doi: 10.1186/1129-2377-15-10 (PMC3942071; doi:10.1186/1129-2377-15-10)

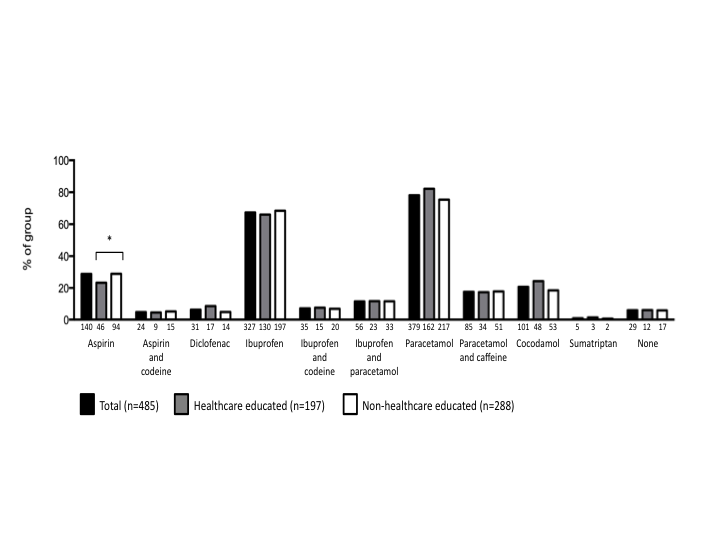

Supplement: Additional file 2: Figure S1 — Medication usage between the two groups demonstrates no significant changes apart from the use of aspirin. Respondents consisted of healthcare educated (n = 197; grey bars) and non-healthcare educated (n = 288; white bars) individuals. Data are demonstrated as percentage of sample selected with the absolute value below each bar. *Indicates p < 0.05. [file 1129-2377-15-10-S2.tiff]
